# The associations of multimorbidity with the sum of annual medical and long-term care expenditures in Japan

**DOI:** 10.1186/s12877-019-1057-7

**Published:** 2019-03-07

**Authors:** Takahiro Mori, Shota Hamada, Satoru Yoshie, Boyoung Jeon, Xueying Jin, Hideto Takahashi, Katsuya Iijima, Tatsuro Ishizaki, Nanako Tamiya

**Affiliations:** 1 Health Services Research & Development Center, University of Tsukuba, Japan, 1-1-1 Tenno-dai, Tsukuba, Ibaraki, 305-8575 Japan; 2 Department of Health Services Research, Faculty of Medicine, University of Tsukuba, Japan, 1-1-1 Tenno-dai, Tsukuba, Ibaraki, 305-8575 Japan; 3 Department of General Internal Medicine, Eastern Chiba Medical Center, Japan, 3-6-2 Okayamadai, Togane, Chiba, 283-8686 Japan; 4 Research Department, Institute for Health Economics and Policy, Association for Health Economics Research and Social Insurance and Welfare, Japan, No.11 Toyo-Kaiji Bldg, 1-5-11 Nishi-Shimbashi, Minato-ku, Tokyo, 105-0003 Japan; 5 Institute of Gerontology, The University of Tokyo, Japan, Faculty of Engineering Bldg.8.,7F. 7-3-1 Hongo Bunkyo-ku, Tokyo, 113-8656 Japan; 6 Department of Health Policy and Management, School of Medicine, Keio University, Japan, 35 Shinanomachi, Shinjuku-ku, Tokyo, 160-8582 Japan; 7 Division of Health Service for the Disabled, National Rehabilitation Center, the Republic of Korea, 520 Suyu5-dong, Gangbuk-gu, Seoul, 01022 the Republic of Korea; 8 National Institute of Public Health, Japan, 2-3-6 Minami, Wako, Saitama, 351-0197 Japan; 9 Human Care Research Team, Tokyo Metropolitan Institute of Gerontology, Japan, 35-2 Sakae-cho, Itabashi-ku, Tokyo, 173-0015 Japan

**Keywords:** Long-term care expenditures, Medical expenditures, Multimorbidity, Charlson comorbidity index, Claims data

## Abstract

**Background:**

The occurrence of multimorbidity (i.e., the coexistence of multiple chronic diseases) increases with age in older adults and is a growing concern worldwide. Multimorbidity has been reported to be a driving factor in the increase of medical expenditures in OECD countries. However, to the best of our knowledge, there is no published research that has examined the associations between multimorbidity and either long-term care (LTC) expenditure or the sum of medical and LTC expenditures worldwide. We, therefore, aimed to examine the associations of multimorbidity with the sum of medical and LTC expenditures for older adults in Japan.

**Methods:**

Medical insurance claims data for adults ≥75 years were merged with LTC insurance claims data from Kashiwa city, a suburb in the Tokyo metropolitan area, for the period between April 2012 and September 2013 to obtain an estimate of medical and LTC expenditures. We also calculated the 2011 updated and reweighted version of the Charlson Comorbidity Index (CCI) scores. Then, we performed multiple generalized linear regressions to examine the associations of CCI scores (0, 1, 2, 3, 4, or ≥ 5) with the sum of annual medical and LTC expenditures, adjusting for age, sex, and household income level.

**Results:**

The mean sum of annual medical and LTC expenditures was ¥1,086,000 (US$12,340; *n* = 30,042). Medical and LTC expenditures accounted for 66 and 34% of the sum, respectively. Every increase in one unit of the CCI scores was associated with a ¥257,000 (US$2920); 95% Confidence Interval: ¥242,000, 271,000 (US$2750, 3080) increase in the sum of the expenditures (*p* < 0.001; *n* = 29,915).

**Conclusions:**

Using a merged medical and LTC claims dataset, we found that greater CCI scores were associated with a higher sum of annual medical and LTC expenditures for older adults. To the best of our knowledge, this is the first study to examine the associations of multimorbidity with LTC expenditures or the sum of medical and LTC expenditures worldwide. Our study indicated that the economic burden on society caused by multimorbidity could be better evaluated by the sum of medical and LTC expenditures, rather than medical expenditures alone.

**Electronic supplementary material:**

The online version of this article (10.1186/s12877-019-1057-7) contains supplementary material, which is available to authorized users.

## Background

A steady increase in health-related spending has been a great concern in the Organisation for Economic Co-operation and Development (OECD) countries [1]. In Japan, the annual medical expenditures in 2016 were approximately ¥42.1 trillion (approximately US$372 billion per the 2016 exchange rate) [2, 3], and the annual long-term care (LTC) expenditures in 2016 were approximately ¥10.0 trillion (approximately US$88 billion) [4]. These expenditures are projected to further increase with a rapidly aging society, as is the case with many OECD countries [1, 5]. Therefore, it is essential to identify the factors associated with the increase in these expenditures to develop strategies to ensure sustainable medical and LTC systems.

The occurrence of multimorbidity (i.e., the coexistence of multiple chronic diseases) increases with age in older adults. Multimorbidity is a growing concern worldwide, as it has been reported to be associated with functional decline, decreased quality of life, and possibly even higher mortality [6]. Furthermore, multimorbidity has been reported to be a driving factor in the increase of medical expenditures in OECD countries [7, 8]. In Japan, however, to the best of our knowledge, no population-based study has been published that examined the associations of overall multimorbidity with medical expenditures. While one study included comorbidity as one of the covariates, its population was limited to cancer patients at the end of life [9]. There also have been a few studies that have focused on the associations between specific medical conditions (e.g., hypertension) and medical expenditures [10, 11] but not the associations between overall multimorbidity and medical expenditures.

Further, to the best of our knowledge, no published research has examined the associations between overall multimorbidity and either LTC expenditures or the sum of medical and LTC expenditures worldwide. Mandatory public LTC insurance systems have been introduced in some OECD countries, such as Germany, the Netherland, and South Korea [12, 13]. While there have been studies published in these countries that examined the associations between various medical conditions and LTC expenditures, these studies did not examine the associations of overall multimorbidity with LTC expenditures. For instance, a study from the Netherlands studied the selected diseases separately rather than multimorbidity [14]. Similarly, a study conducted in Germany examined the associations between dementia and both medical and long-term care expenditures [15]. Also, a South Korean study examined the associations between dementia, stroke, both dementia and stroke, and those who had neither dementia nor stroke and LTC expenditures [16].

Japan developed its universal medical insurance system in 1961 and implemented a new scheme in 2008. Every individual who is at least 75 years old, except for those receiving public assistance, is eligible for the Late-Stage Medical Care System for the Elderly, which replaces the medical insurance coverage for those who are less than 75 years old [17, 18]. Coverage by the Late-Stage Medical Care System for the Elderly includes services provided by medical professionals (e.g., doctors, nurses, various therapists), diagnostic tests, prescriptions, surgery, and anesthesia. In addition to the universal medical insurance system, Japan also launched the mandatory public LTC insurance system in 2000. Those aged 65 and older, as well as those aged between 40 and 64 years with specific aging-related diseases, are eligible for the services, including not only institutional care (e.g., long-term admission or short-term stay in a LTC facility) but also community- and home-based care (e.g., adult day care, outpatient rehabilitation, home help, or home-visit nursing) [12, 19].

The main purpose of our study was to examine the associations between baseline multimorbidity and annual medical expenditures, LTC expenditures, and the sum of both medical and LTC expenditures. We hypothesized that greater multimorbidity was associated with both higher medical and LTC expenditures, and hence the sum of both types of expenditures. We also examined the associations between multimorbidity and the level of LTC required. In Japan, the level of LTC required consists of seven levels (Support Levels 1–2, and Care Levels 1–5), with Support Level 1 representing the lowest level and Care Level 5 representing the highest level of requirement for LTC. The level of LTC required is assessed and approved systemically and reevaluated periodically (at least every two years) [12]. For greater detail on the evaluation process for LTC services in Japan, please refer to Tamiya and colleagues’ article [11]. We hypothesized that greater multimorbidity would be associated with a higher level of LTC required.

## Methods

### Data source and participants

**Fig. 1:**
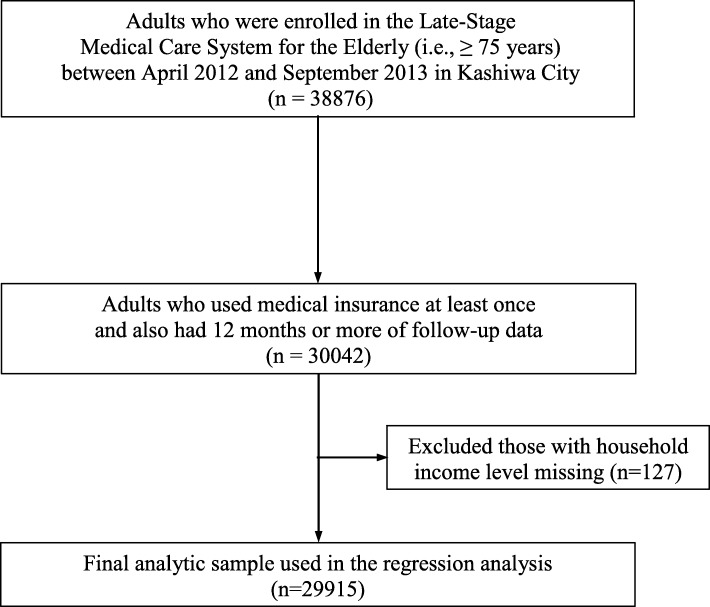
Flowchart of the study’s sample population

We obtained medical insurance claims data of the Late-Stage Medical Care System for the Elderly (i.e., adults ≥75 years) and LTC insurance claims data from the municipal government of Kashiwa City: a suburb in the Tokyo metropolitan area. Kashiwa City had a population of approximately 405,000 in 2012 with 8.7% of the population being at least 75 years old in October 2012 [20]. Insurance claims data for the period between April 2012 and September 2013 were available for analysis at the time of this study. In this study, we included those who were enrolled in the Late-Stage Medical Care System for the Elderly (i.e., adults ≥75 years) between April 2012 and September 2013 in Kashiwa city (Fig. 1).

The datasets did not contain personally identifiable information; however, the same dummy ID numbers were assigned to each individual in both medical and LTC claim datasets. We merged the medical insurance claims data for those aged ≥75 years with the LTC insurance claims data at an individual level using these dummy ID numbers. We could not obtain medical insurance claims data for those covered by public assistance from the city; therefore, we did not include these in this analysis.

We included medical insurance claims data from both Diagnosis Procedure Combination/Per-Diem Payment Systems (DPC/PDPS) and non-DPC/PDPS. DPC/PDPS was introduced in Japan in 2003, and it offers a case-mix payment system for acute inpatient care according to diagnoses and procedures [20].

### Measurements

From the medical insurance dataset, we obtained data regarding medical expenditures, including inpatient/outpatient medical care payments and medication costs, but did not include dental care, along with Charlson Comorbidity Index (CCI) scores, age (e.g., the 5-year ranges of birth years [e.g., 1930–34]), and sex. From the LTC insurance dataset, we obtained data on LTC expenditures, LTC insurance premiums, and the level of LTC required.

To obtain the sum of medical and LTC expenditures for the 12 months period, we totaled each month’s expenditures, from and including the month, in which medical insurance had been used for the first time since April 2012 (i.e., the index month). The total amount of expenditures included both insurance reimbursements and co-payments for the covered services. The expenditures were presented both as Japanese yen (¥) and U.S. dollars ($; the mean exchange rate from April 2012 to September 2013 was 88 Japanese yen to $1) [21], unless otherwise specified.

We excluded those who did not use any medical insurance during the period of analysis. We also excluded from analysis those who had less than 12 months of follow-up data (Fig. 1) because we did not have comprehensive information regarding when they either obtained or lost medical insurance. Additionally, we did not have information regarding whether an individual lived in Kashiwa City as of April 2012, or if he or she had moved from the city during the study period. Finally, we did not have reliable information regarding all deaths that occurred during the period; the medical insurance dataset provided information regarding death during hospitalization but did not include deaths that occurred outside of hospitals.

We calculated CCI scores, which were previously used as a proxy for multimorbidity to predict future medical expenditures [22, 23]. We used the 2011 updated and reweighted version of the original CCI scores because these updated scores have been validated in a Japanese national administrative dataset to predict in-hospital mortality [24].

To define the multimorbidity in this study, we obtained the CCI scores for the inclusive period within the three months after the index month in which medical insurance had been used for the first time since April 2012 (i.e., if the index month was June 2012, the CCI scores were calculated based on the claims data from June 2012 to August 2012). We chose a period of three months because, in Japan, neither medication prescriptions beyond a three-month period nor refills are allowed, which generally requires patients to visit his or her physician at least every three months for chronic conditions.

We identified conditions based on the International Classification of Diseases, Tenth Revision (ICD-10) codes that were provided in the medical insurance dataset [25]. As the ICD-10 codes in this dataset were only represented with integers (e.g., I11 for hypertensive heart disease instead of I11.0 for hypertensive heart disease with congestive heart failure), we also used the disease codes in the medical insurance dataset, which provided more detailed information. The conditions with a “suspicious” flag in the medical claims dataset, which suggested that the diagnoses were placed to justify diagnostic procedures, were not included in the calculation of CCI scores. We treated the CCI variable as a discrete variable and assigned scores that were greater than 5 to the category of 5 (i.e., scores of 0, 1, 2, 3, 4, ≥ 5). This scoring rationale was based on one used in a previous study [24] and on the distribution of CCI scores in our study (i.e., the proportion of CCI scores ≥6 was only 3%; Table 1).

**Table 1:** Descriptive characteristics of the study population (*N* = 30,042^a^)

	n (%)
Charlson Comorbidity Index^b^	
0	13,732 (45.7%)
1	3167 (10.5%)
2	7199 (24.0%)
3	2254 (7.5%)
4	1942 (6.5%)
5	787 (2.6%)
6	540 (1.8%)
7	216 (0.7%)
8	106 (0.4%)
9	52 (0.2%)
≥10	47 (0.2%)
Birth year (ages as of January 1, 2012)	
1900–1914 (97–111)	162 (0.5%)
1915–1919 (92–96)	862 (2.9%)
1920–1924 (87–91)	2769 (9.2%)
1925–1929 (82–86)	6201 (20.6%)
1930–1934 (77–81)	11,137 (37.1%)
1935–1939 (72–76)^c^	8911 (29.7%)
Sex	
Men	12,561 (41.8%)
Women	17,481 (58.2%)
Household income level (*n* = 29,915)	
Low-income group	8823 (29.5%)
Middle-to-high income group	21,092 (70.5%)
Level of long-term care required	
Not required	22,657 (75.4%)
Support level 1	586 (2.0%)
Support level 2	787 (2.6%)
Care level 1	1574 (5.2%)
Care level 2	1558 (5.2%)
Care level 3	1143 (3.8%)
Care level 4	918 (3.1%)
Care level 5	819 (2.7%)

^a^Unless otherwise specified

^b^The 2011 updated and reweighted version

^c^We included only those individuals who were enrolled in the Late-Stage Medical Care System for the Elderly (i.e., adults ≥75 years) in this analysis

We followed the original definitions of all of the diseases or medical conditions as much as possible [22]. However, in some instances, the diseases or medical conditions we included did vary from the original definitions. For details regarding these variations, please refer to the information presented in Additional file 1.

The age variable was divided into two groups (i.e., the 5-year ranges for birth year < 1920–1924 or > 1925–1930, which corresponded to age ≥ 87 years or age ≤ 86 years, respectively, as of January 1, 2012). As a proxy for socioeconomic status, we used the LTC insurance premiums category, which were based on household income. The premiums category was divided into two groups, a low-income group for those participants (along with their family members) who were exempt from residents’ taxation and a middle-to-high income group for the remaining participants [26].

### Analysis

We first obtained the descriptive characteristics of the sample, 12 months of medical expenditures, 12 months of LTC expenditures, and the sum of these medical and LTC expenditures. Next, we performed multiple generalized linear regressions with family gamma and long-link function to examine the associations between the CCI scores and 1) the sum of medical and LTC expenditures, 2) medical expenditures alone, and 3) LTC expenditures alone, adjusting for age, sex, and household income level [9]. In addition, to examine if the CCI scores were associated with expenditures within the same level of LTC required (i.e., LTC not required, Support Levels 1–2, and Care Levels 1–5), we further stratified by each level of LTC required and performed multiple generalized linear regressions with family gamma and long-link function to examine the associations between the CCI scores and these expenditures, adjusting for the same covariates. We then performed multiple ordinary logistic regressions to examine the associations of the CCI scores with the 7 levels of LTC required (reference: LTC not required), adjusting for age, sex, and household income level. After performing these regressions, we used the delta method to obtain the predicted coefficients of medical, LTC, and the sum of both expenditures, probabilities, and the 95% Confidence Intervals (CI). All statistical tests were two-tailed with a significant level of *p* < 0.05 using the STATA Version 14.2 (StataCorp LP, College Station, TX, USA).

## Results

Of 30,042 individuals, the descriptive statistics showed that the mean and median scores of CCI were 1.42 and 1, respectively (Table 1). For those who required LTC (*n* = 7385), the mean and median scores were 2.01 and 2, respectively, while for those who did not required LTC (*n* = 22,657), the mean and median scores were 1.22 and 0, respectively. Men and women accounted for 42 and 58%, respectively. Ages were presented in five-year ranges based on birth year, and 88% fell into the ranges of 1925–1929, 1930–1934, and 1934–39, which corresponds to 88% of individuals being 86 years old or younger as of January 1, 2012 (Table 1). The mean medical expenditures, LTC expenditures, and the sum of these expenditures for 12 months were ¥ 716,000 ($8140), ¥370,000 ($4200), and ¥1,086,000 ($12,340), respectively (*n* = 30,042; Fig. 1, Table 2). Medical and LTC expenditures accounted for 66 and 34%, respectively.

**Table 2:** Medical and long-term care expenditures for a 12-month period (*n* = 30,042)

The sum of medical and long-term care	¥1,086,000 (US$ 12,340)
Medical	¥716,000 (US$ 8140)
Inpatient care	¥322,000 (US$ 3660)
Outpatient care	¥394,000 (US$ 4480)
Long-term care	¥370,000 (US$ 4200)

¥88 was equivalent to $1, which was the mean exchange rate from April 2012 to September 2013

Multiple generalized linear regressions showed that CCI scores were associated with higher amounts of each expenditure, and therefore the sum of both types of expenditures (*p* < 0.001, *n* = 29,915; Table 3; Fig. 1). For example, every increase in one unit of CCI scores (0, 1, 2, 3, 4, or ≥ 5) was associated with a ¥257,000 ($2920) increase in the sum of both medical and LTC expenditures (95% CI [¥242,000, ¥271,000] or 95% CI [$2750, $3080]). A simple generalized linear regression without adjusting for these covariates (i.e., age, sex, and household income level) showed ¥261,000 ($2970) increase in the sum of the expenditures (95% CI [¥247,000, ¥275,000] or 95% CI [$2810, $3130]; *p* < 0.001). After being stratified by the level of LTC required, CCI scores were no longer associated with LTC expenditure in any of the level of LTC required (*p* values ranging from 0.096 to 0.792, Table 3).

**Table 3:** The associations of medical and long-term care expenditures with the Charlson Comorbidity Index (CCI) scores^a^ using generalized linear regressions (*n* = 29,915)

	The sum of medical and long-term care expenditures			Medical expenditures			Long-term care expenditures		
	Coefficient	*P* value	95% CI	Coefficient	*P* value	95% CI	Coefficient	*P* value	95% CI
Total (*n* = 29,915)									
CCI scores^a^	25.7 ($2920)	< 0.001	24.2, 27.1 ($2750, 3080)	15.7 ($1780)	< 0.001	14.7, 16.6 ($1670,1890)	12.0 ($1360)	< 0.001	10.5, 13.5 ($1200,1530)
Care not required (*n* = 22,657)									
CCI scores^a^	13.8 ($1570)	< 0.001	12.8, 14.9 ($1460,1690)	13.8 ($1570)	< 0.001	12.8, 14.9 ($1460, 1690)	n/a	n/a	n/a
Support Level 1 (*n* = 586)									
CCI scores^a^	9.3 ($1060)	< 0.001	4.7, 13.9 ($540, 1570)	9.3 ($1050)	< 0.001	4.6, 13.9 ($530,1580)	−0.3 (−$40)	0.520	−1.3, 0.6 (−$140,70)
Support Level 2 (*n* = 787)									
CCI scores^a^	5.6 ($640)	0.008	1.5, 9.8 ($170, 1110)	6.3 ($720)	0.002	2.4, 10.3 ($270,1170)	−1.3 (−$140)	0.096	−2.8, 0.2 (−$320,30)
Care Level 1 (*n* = 1574)									
CCI scores^a^	6.0 ($680)	0.003	2.1, 9.9 ($240, 1120)	5.7 ($650)	0.001	2.4, 9.0 ($270,1030)	0.5 ($60)	0.654	−1.8, 2.9 (−$200,330)
Care Level 2 (*n* = 1558)									
CCI scores^a^	10.3 ($1180)	< 0.001	5.7, 15.0 ($650, 1700)	9.9 ($1120)	< 0.001	5.5, 14.3 ($630,1620)	0.4 ($50)	0.792	−2.5, 3.3 (−$290,370)
Care Level 3 (*n* = 1143)									
CCI scores^a^	14.7 ($1680)	< 0.001	9.3, 20.1 ($1060, 2290)	13.3 ($1520)	< 0.001	8.0, 18.6 ($910,2120)	0.8 ($90)	0.685	−3.2, 4.8 (−$360,550)
Care Level 4 (*n* = 918)									
CCI scores^a^	14.6 ($1650)	< 0.001	8.3, 20.8 ($940,2370)	16.3 ($1850)	< 0.001	9.3, 23.3 ($1050,2650)	−1.6 (−$190)	0.490	−6.3, 3.0 (−$720, 340)
Care Level 5 (*n* = 819)									
CCI scores^a^	18.9 ($2140)	< 0.001	13.1, 24.6 ($1490,2800)	18.1 ($2060)	< 0.001	10.9, 25.3 ($1240,2870)	1.6 ($180)	0.520	−3.2, 6.3 (−$360,720)

*CI* Confidence Interval

^a^The 2011 updated and reweighted version (0, 1, 2, 3, 4, ≥ 5)

¥88 was equivalent to $1. The results are presented in units of 10,000 yen (US$)

The tables show the total and the stratification by the level of long-term care required

**Fig. 2:**
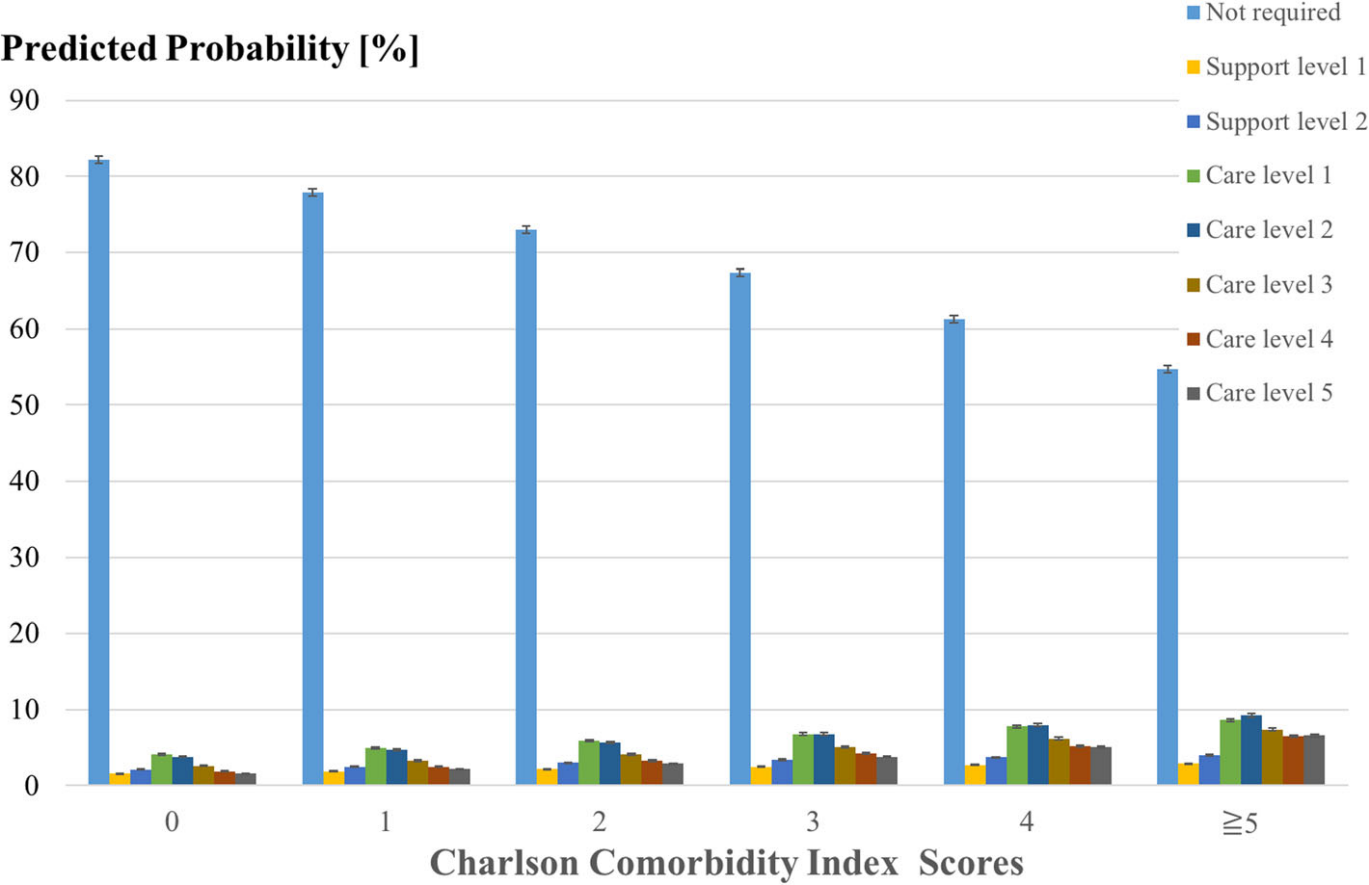
The predicted probabilities for long-term care use (*n* = 29,915). The level of long-term care required consists of seven levels (Support Levels 1–2, and Care Levels 1–5), with Support Level 1 representing the lowest level and Care Level 5 representing the highest level of requirement for long-term care. Error bars represent the 95% Confidence Intervals. Results were obtained by ordinal logistic regressions, adjusting for age, sex, and household income level.

In the ordinal multiple logistic regression, greater CCI scores were also associated with higher levels of LTC requirements (*p* < 0.001; *n* = 29,915). For those with CCI scores of 0, the predicted probabilities of not requiring LTC or having Care Level 5 were 82.2% (95% CI [81.7, 82.7]) or1.6% (95% CI [1.5, 1.7]), respectively. On the other hand, for those with CCI scores of 5 or higher, the predicted probabilities of not requiring LTC or having Care Level 5 were 54.7% (95% CI [53.3, 56.1]) or 6.6% (95% CI [6.1, 7.1]), respectively (Fig. 2).

## Discussion

In this population-based study using merged medical and LTC claims data, greater CCI scores were associated not only with higher medical expenditures, but also with higher LTC expenditures, and hence with the sum of both expenditures. Greater CCI scores were also associated with higher levels of LTC required. Our study, therefore, suggested that the economic burden on society caused by multimorbidity can be evaluated better by considering both medical and LTC expenditures, rather than medical expenditures alone.

To the best of our knowledge, this is the first study to examine the associations between multimorbidity and medical expenditures in Japan. Our study indicated that greater multimorbidity was associated with higher medical expenditures, which was consistent with the results from other OECD countries [7, 8, 23]. In addition, in our study, those who required LTC had higher mean and median CCI scores compared with those who did not require LTC.

More importantly, this is the first study, worldwide, to examine the associations of multimorbidity with LTC expenditures. In Japan, although there have been at least two published studies that analyzed the determinants of LTC expenditures [27, 28], and one study that included both LTC and medical expenditures [29], these studies did not include multimorbidity as either the main predictor variable or as one of the covariates. Previous research appeared to have used only LTC insurance claims data [27, 28] or annual assessments and biennial surveys [29], which did not contain the medical information necessary to identify multimorbidity. In this research, we took a novel approach in which we obtained medical and LTC claims data of the entire city and merged them at an individual level. This method enabled us to add LTC information to medical claims data, add CCI scores to LTC claims data, and to calculate the sum of medical and LTC expenditures.

We found that greater CCI scores were also associated with higher levels of LTC requirements. Within the same level of LTC, however, multimorbidity was not significantly associated with LTC expenditures. Given that higher levels of LTC have higher limits for benefits paid for services per month [12], we concluded that greater comorbidity was associated with the higher levels of LTC, leading to greater LTC expenditures. We further explored and confirmed that higher levels of LTC were associated with higher LTC expenditures in this study (*p* < 0.001, data not shown). These results implied that unmet LTC needs might exist, in which those who required higher levels of LTC services could not receive sufficient services due to the limits of the benefits paid for the services. Once their LTC levels became higher, they could obtain more services as the upper limits of the benefits increased. The existing care situation (e.g., family environment) is not considered when determining the LTC levels, potentially creating an unmatched supply and demand for LTC services [30, 31]. An alternative explanation is that the supply might induce the demand. In other words, care managers who play a primary role in determining what services are provided by the LTC system may use the upper limits of the benefits regardless of the services an individual needed.

Our research has several limitations. First, it was challenging to obtain precise CCI scores based on the available claims data, as, for example, the original definition of several conditions requires specific values that were not available on the claims data. Additionally, tentative diagnoses were sometimes given to justify diagnostic procedures in a fee-for-service system. These diagnoses were supposed to be tagged with a “suspicious” flag in the medical claims data and were excluded from our CCI score calculations. There also was a possibility that tentative or false diagnoses were placed, so that medical insurance would cover an individual’s prescriptions. Further, there was a possibility that some diagnoses were left out of the dataset because they were not related to any procedure reimbursements or medication prescriptions. We, however, made our best effort to obtain CCI scores by using claims data to avoid these limitations. Secondly, CCI scores did not fully assess the severity of the participants’ chronic diseases, which may play an important role in the expenditures. Thirdly, the dataset did not provide precise information as to when a participant obtained or lost medical insurance eligibility; because of this, we only included those with a minimum of 12 months of follow-up data. This approach was likely to contribute to an underestimate of the expenditures, as it has been reported that medical expenditures tend to increase in the last months of life [32, 33]. Indeed, our mean for medical expenditures was ¥ 716,000 ($8140), which was lower than the actual amount estimated by the local government’s report (which was approximately ¥ 770, 000 ($8750) in 2013) [34]. In addition, there also was a possibility that we might have excluded more severely ill individuals (i.e., those with higher CCI scores), who died within 12 months from the index month, leading to underestimating the expenditures. Finally, we did not include those who received public welfare due to the unavailability of the data, which likely led to an underrepresentation of the low-income group.

However, our study does have an important strength. This is the first study to show that greater multimorbidity was associated not only with medical expenditures but also with LTC expenditures and hence with the sum of both expenditures. Multimorbidity is a growing concern in aging society and has been associated with multiple undesirable outcomes [6]. Our results suggested that multimorbidity also imposed an economic burden on society by increasing not only medical but also LTC expenditures. Therefore, effective strategies to target for reducing the prevalence of multimorbidity could provide not only medical and functional but also economic benefits for individuals and society. As the steady increase in health-related spending has been a great concern in OECD countries, our results are meaningful not only for Japan but also for other countries.

## Conclusions

In this population-based study using merged medical and LTC claims data in Japan, we found that multimorbidity was positively associated with the sum of medical and LTC expenditures in older adults. These results demonstrated that it is important to sum both medical and LTC expenditures to estimate the total economic burden on society associated with multimorbidity.

## Additional file

Additional file 1: The variations of the diseases or medical conditions from the original definitions to obtain Charlson Comorbidity Index scores. To obtain Charlson Comorbidity Index scores, we followed the original definitions of all of the diseases or medical conditions as much as possible. However, in some instances, the diseases or medical conditions we included did vary from the original definitions. Details regarding these variations were presented in Additional file 1. (DOCX 13 kb)

## Abbreviations

CCI: Charlson Comorbidity Index
CI: Confidence Interval
DPC/PDPS: Diagnosis Procedure Combination/Per-Diem Payment Systems
ICD-10: International Classification of Diseases, Tenth Revision
LTC: Long-term care
OECD: Organisation for Economic Co-operation and Development
