# Supplementary material for: The associations of multimorbidity with the sum of annual medical and long-term care expenditures in Japan
Source: BMC Geriatr. 2019 Mar 7;19:69. doi: 10.1186/s12877-019-1057-7 (PMC6404301; doi:10.1186/s12877-019-1057-7)
Supplement: Supplementary file 1 — The variations of the diseases or medical conditions from the original definitions to obtain Charlson Comorbidity Index scores. To obtain Charlson Comorbidity Index scores, we followed the original definitions of all of the diseases or medical conditions as much as possible. However, in some instances, the diseases or medical conditions we included did vary from the original definitions. Details regarding these variations were presented in Additional file 1. (DOCX 13 kb) [file 12877_2019_1057_MOESM1_ESM.docx]

**Additional file 1**

Those with an untreated thoracic or abdominal aneurysm (6cm or more) were categorized has having peripheral vascular disease, but the dataset did not contain information about the diameters of the aneurysms, so in this study, we have decided to include all aneurysms.

The original definition of cerebrovascular disease included patients with a history of a cerebrovascular accident with minor or no residual effects, but the dataset did not provide precise information regarding the status of the residual effects. We therefore decided that patients with paralysis were not categorized as having a cerebrovascular disease, although there was a possibility that paralysis might be due to diseases or conditions other than cerebrovascular disease.

For rheumatoid disease, we also included Sjögren's syndrome, vasculitis, and Bechet disease, which were not included in the original definition for rheumatoid disease. In the original definition, only moderate to severe rheumatoid arthritis (RA) was included, but there was no data regarding the severity of RA, so we decided to include all instances of RA.

Also, the original definition for moderate renal disease only included patients with serum creatinine levels greater than 3mg/dl but, as the dataset did not contain the creatinine levels, we included chronic kidney diseases (CKD) stage G4 (with a corresponding GFR of 15-29 mL/min/1.73m^2^) and worse.

Finally, in the original definition, mild liver disease included cirrhosis without portal hypertension. In the dataset, however, we consider those with cirrhosis as categorized with either moderate or severe liver disease because precise information regarding the status of portal hypertension was not available in the dataset. We also included fatty liver and elevated liver enzymes as mild liver disease even though the original definition of mild liver disease consisted of cirrhosis without portal hypertension or chronic hepatitis.
